# Cumulative effects of prenatal-exposure to exogenous chemicals and psychosocial stress on fetal growth: Systematic-review of the human and animal evidence

**DOI:** 10.1371/journal.pone.0176331

**Published:** 2017-07-12

**Authors:** Hanna M. Vesterinen, Rachel Morello-Frosch, Saunak Sen, Lauren Zeise, Tracey J. Woodruff

**Affiliations:** 1 Program on Reproductive Health and the Environment, University of California, San Francisco, United States of America; 2 Department of Environmental Science, Policy and Management and School of Public Health, University of California, Berkeley, United States of America; 3 Department of Preventive Medicine, University of Tennessee Health Science Center, Memphis, United States of America; 4 California Environmental Protection Agency Office of Environmental Health Hazard Assessment, Oakland, United States of America; Stony Brook University, Graduate Program in Public Health, UNITED STATES

## Abstract

**Background:**

Adverse effects of prenatal stress or environmental chemical exposures on fetal growth are well described, yet their combined effect remains unclear.

**Objectives:**

To conduct a systematic review on the combined impact and interaction of prenatal exposure to stress and chemicals on developmental outcomes.

**Methods:**

We used the first three steps of the Navigation Guide systematic review. We wrote a protocol, performed a robust literature search to identify relevant animal and human studies and extracted data on developmental outcomes. For the most common outcome (fetal growth), we evaluated risk of bias, calculated effect sizes for main effects of individual and combined exposures, and performed a random effects meta-analysis of those studies reporting on odds of low birthweight (LBW) by smoking and socioeconomic status (SES).

**Results:**

We identified 17 human- and 22 animal-studies of combined chemical and stress exposures and fetal growth. Human studies tended to have a lower risk of bias across nine domains. Generally, we found stronger effects for chemicals than stress, and these exposures were associated with reduced fetal growth in the low-stress group and the association was often greater in high stress groups, with limited evidence of effect modification. We found smoking associated with significantly increased odds of LBW, with a greater effect for high stress (low SES; OR 4.75 (2.46–9.16)) compared to low stress (high SES; OR 1.95 (95% CI 1.53–2.48)). Animal studies generally had a high risk of bias with no significant combined effect or effect modification.

**Conclusions:**

We found that despite concern for the combined effects of environmental chemicals and stress, this is still an under-studied topic, though limited available human studies indicate chemical exposures exert stronger effects than stress, and this effect is generally larger in the presence of stress.

## Introduction

Both prenatal psychosocial stress and exposure to exogenous environmental chemicals have been found to be independently associated with increased risk of adverse pregnancy outcomes, including low birth weight, gestational hypertension and preterm birth. Significant stress-related risk factors which have been reviewed systematically and found to affect pregnancy and developmental outcomes include: racial disparities and discrimination [1, 2], poverty [3], and psychological distress such as depression or anxiety [4]. Moreover, relaxation techniques during pregnancy have been found to improve pregnancy outcomes [5]. Similarly, there is a separate body of evidence finding associations between the adverse pregnancy outcomes and exogenous chemicals such as lead [6, 7], pesticides [8, 9] and cigarette-smoke [10, 11]. While there is robust evidence of an association between either psychosocial stress *or* chemical exposures and adverse pregnancy outcomes, the effects of their cumulative or combined exposure, often referred to as a form of “double jeopardy” [12], remain unclear.

Adverse pregnancy outcomes such as low birth weight, gestational hypertension and preterm birth are associated with greater perinatal mortality and morbidity, neurodevelopmental disabilities and other complications which span a lifetime including adult onset cardiovascular and metabolic disease [13–17]. Preterm birth has increased steadily over the last twenty years and only recently plateaued. However both preterm birth and low birth weight are still relatively prevalent at 11% and 8% respectively in 2012 [18], with consequential economic and societal burden [19]. Thus there remains an urgent need to understand all of the risk factors that contribute to these complications.

The majority of epidemiologic studies assess the impact of individual chemical exposures, or groups of similar exposures, such as multiple traffic-related air pollutants; however pregnant women are exposed to a plethora of both exogenous environmental chemicals at varying doses [20], and psychosocial stressors [21]. The importance of the cumulative effects of chemical and stress exposures has been identified as a key research need [22, 23] and while there is general acknowledgement that there is likely to be a cumulative effect, to our knowledge there has been no systematic review of the human and animal evidence that holistically evaluates scientific findings regarding whether combined exposures to chemicals and stress are worse than either alone, and if there interactive effects. Advancing our knowledge in this area will enhance understanding about the complexity of risk factors for adverse pregnancy outcomes and inform interventions, such as whether targeting one or the other during the prenatal period is sufficient to reduce the likelihood of adverse health impacts.

We conducted a systematic review of the literature assessing the impact of psychosocial stress and environmental chemicals on developmental outcomes in humans and mammals. Our aim was to evaluate whether combined exposure to both prenatal stress and exogenous chemicals exerted a stronger effect on development outcomes compared to either exposure alone, and, if there is a combined effect, whether there is an interaction. Specifically we sought to: (1) identify all of the relevant human and non-human mammalian studies; (2) extract relevant outcome data and rate the quality of studies; (3) analyze and report the magnitude of the main effects of individual and combined exposures to chemicals and stress and their interaction.

## Methods

To assess the scope of the literature on this topic, we adapted and applied the first three steps of the Navigation Guide systematic review methodology for environmental health [24] to assess the strength of evidence for the combined effect of prenatal stress and chemical exposure on fetal growth. We conducted our systematic review as outlined *a priori* in a protocol which is available online (http://www.dcn.ed.ac.uk/camarades/research.html#protocols) and summarized below. The PRISMA checklist can be found in S1 Table. For the scope of this we review, we focused on the first three steps of the Navigation Guide in order to systematically identify the current literature on this topic, assess the internal validity of the literature, and evaluate potential combined and interactive effects across studies. We defined stress as markers or surrogate indicators of potential prenatal stress exposures that that could result in adverse developmental outcomes in offspring.

### Step 1. Specify the study question

We used a ‘PECO’ (Participants, Exposure, Comparator, Outcome) aid to outline the study question [24]. Our aim was to answer the following questions: (1) ‘does exposure to both prenatal stress and exogenous chemicals result in a worsening of developmental outcomes compared to either exposure alone’; and (2) ‘is there an interaction between prenatal exposure to exogenous chemicals and stress on developmental outcomes’. To answer to these questions we evaluated the combined effect of prenatal stress and exogenous chemicals, with a goal of assessing whether they were additive (question 1), and then whether they were multiplicative (question 2).

**P**opulation: Humans or animals (non-human mammals; hereafter referred to simply as “animals”) that are studied during the reproductive or developmental time period, specifically before and/or during pregnancy for females or during development for fetuses.

**E**xposure(s): (1) Any quantified exogenous chemical exposure before or during pregnancy, and in the same study (2) exposure to chronic psychosocial stress in humans, or equivalent in animals, during the same developmental period. We defined the universe of exogenous chemical exposures as those that are generally addressed by USEPA, and include manufactured chemicals and chemical byproducts (e.g. air pollution) and smoking. A list of chemical exposure terms is included in our protocol in the search term. We defined stress to include major life events, as well as extrinsic sources of stress related to individual-level or place-based measures of material deprivation, poverty, and other indicators of socioeconomic status (SES) or social position.

For our review, we considered three exposure groups (Table 1):

1. Humans exposed to high levels (as defined using the method outlines in the section “Exposure and comparator categorisation”) of both the exogenous chemical and psychosocial stress or animals administered a chemical and subjected to a stress condition;
2. Humans exposed to high levels of the exogenous chemical and low levels of stress, or animals administered a chemical and not subjected to the stress condition;
3. Humans exposed to low levels of the exogenous chemical and high stress, or animals untreated or administered vehicle and subjected to the stress condition.

**Table 1:** A summary of the relationships we assessed between chemical or stress or both exposures and the comparisons group.

	Group	Exposure		Comparator (Group 4)		Main effect investigated
		Chemical	Stress	Chemical	Stress	
**Human studies**	**#1a**	High	High	Low	Low	Main effect of combined exposure
	**#2a**	High	Low			Main effect of chemical
	**#3a**	Low	High			Main effect of stress
**Animal studies**	**#1b**	Yes	Yes	No	No	Main effect of combined exposure
	**#2b**	Yes	No			Main effect of chemical
	**#3b**	No	Yes			Main effect of stress

**C**omparators: Humans exposed to low levels of both the exogenous chemical *and* psychosocial stress during the same developmental period, or animals not treated with the chemical and not subjected to the stress condition (vehicle controls).

**O**utcomes: Measures of fetal growth including birth weight, and/or measures of size such as birth length, head or abdominal circumference; preterm birth and/or gestational age; developmental outcomes within the first twelve years (humans) or before adulthood (animals).

### Step 2. Select the evidence

#### Search methods

We used known relevant articles on the effect of either exogenous chemicals or stress to populate a list of relevant Medical Subject Headings (MeSH) which we expanded using the MeSH tree in Pubmed. This yielded a list of key MeSH terms and keywords (see online protocol). We searched four online databases on April 18^th^ 2013 (Pubmed, ISI Web of Science, Biosis Previews, Embase). In addition, we conducted a second search in Pubmed on May 29^th^ 2013 using additional MeSH terms and keywords from relevant articles identified in the first search. Finally, we hand searched the reference list of all studies included in the systematic review to identify additional relevant studies. Our search was not limited by date or language.

#### Study selection criteria

We based our inclusion and exclusion criteria on the study question and the characteristics described in the ‘PECO’ aid. One reviewer (HV) screened titles and abstracts of the search results against the inclusion and exclusion criteria (Table 2) using a custom form in Distiller SR (Evidence Partners; available at http://systematic-review.net). A second reviewer (HS) independently screened 20% with no discrepancies between reviewers. The vast majority did not meet the inclusion criteria. Studies which either met the inclusion criteria or for which eligibility could not be determined from titles and abstracts alone were taken forward to the full text screen. One reviewer performed the full text screen (HV).

**Table 2:** The inclusion and exclusion criteria used to determine study eligibility for inclusion in the systematic review.

Inclusion criteria	Exclusion criteria
Original research articles with human or non-human mammalian subjects; Developmental outcomes reported for the four exposure groups; (1) high exogenous chemical and low stress; (2) low exogenous chemical and high stress; (3) high exogenous chemical and high stress; and (4) low exogenous chemical and low stress; Exposure during or prior to pregnancy; Developmental outcomes within the first twelve years (humans) or prior to adulthood (animals).	Reviews or other non-original research articles; One or more exposure group is not reported; Exposure is not during or prior to pregnancy; Developmental outcomes up to age 12 in humans, or prior to adulthood in animals are not reported. Other (with reason).

#### Data collection

One reviewer (HV) extracted relevant data pertaining to the publication (unique reference ID, first and corresponding authors, year of publication, type of publication (full or meeting abstract), the exposures (the exogenous chemical and stressor, dose or other measure of exposure), and the endpoints (the outcome measure, sample size per group, a measure of central tendency, incidence or odds ratio, a measure of variance (standard deviation (SD), standard error of the mean (SEM) or 95% CI), the sex of the offspring, and the time of assessment relative to day of birth (day zero). For animal studies we additionally extracted data on the route of administration and timing of administration (relative to gestational day zero), the species and strain.

Where data were missing or incomplete we made up to two attempts to obtain the information directly from study authors.

#### Exposure and comparator categorization

For animal studies we took groups administered the chemical and/or a stress condition as an exposed group, and any untreated or vehicle administered group without the stress condition as the comparator group. For human studies we dichotomized stress exposure variables into “high” and “low” as detailed in Table 3, and pooled the means and variances using previously described methods [Chapter 7 from 25].

**Table 3:** A summary of the human stress exposure variables assigned to the “low” and “high” exposure groups. A detailed description of the exposures can be found in the original studies which are listed by citation number.

Exposure Type	Low stress exposure	High stress exposure	References
Race	White	Asian, Middle Eastern, Pacific Islanders, African, “other”	[26]
		Black	[27]
	Non-Hispanic White	Black, Hispanic, Non-Hispanic Asian & Pacific Islanders	[28]
Educational attainment	13+ years	10 to 12 years, 9 years or less	[29]
	>12 years	<12 years	[30]
	11+ years	9–10 years, <8 years	[31]
	“Other” education, university, technical college	High school, primary school	[26]
Socioeconomic status or social class	Professional, Intermediate	Skilled, semi-skilled, partly skilled, unskilled, unemployed	[32, 33]
	High social class	Middle & low social classes	[34]
	Household income >median	Household income <median	[30]
	Neighborhood poverty (% low income families): 0 to 7% & 7 to 14%	Neighborhood poverty (% low income families): 14 to 22%, 22 to 32% & >32%	[28]
	Financial difficulty in affording food: level 1	Financial difficulty in affording food: level 2 & 3	[35]
Maternal mood	High mood	Low mood	[36]
Life events	0 major life events	1 major life events; 2+ major life events	[37]

### Step 3. Assess the risk of bias of each individual study

We used the Navigation Guide risk of bias tool for human and animal studies which has been previously described [38, 39]. Five domains were the same for both human and animal studies: blinding; incomplete outcome data; selective outcome reporting; conflicts of interest; and any other sources of bias. Additionally for human studies we assessed recruitment strategy; exposure assessment and confounding, and for animal studies we assessed sequence generation. Further information on these domains are provided in the study protocol (http://www.dcn.ed.ac.uk/camarades/research.html#protocols). For each domain we assigned a rating of “low risk”, “probably low risk”, “probably high risk” or “high risk”. Definitions of the domains were the same as in [40] and [41].

### Step 4. Quantitative evaluation of effects of combined exposures

We conducted a quantitative analysis on developmental endpoints in humans and animals which were reported in five or more human studies; only outcomes on fetal growth met this criterion. Specifically, we evaluated data from human studies on birth weight, relative change in or odds of low birth weight; and from animal studies on fetal weight, birth weight or postnatal weight. Accordingly, we focused our first study question (above) on fetal growth, which became:

- **Study question 1:** Does exposure to both prenatal stress and exogenous chemicals result in a decrease in growth compared to either exposure alone?

For this question, we focused on evaluating the additive effects of the chemical exposures and psychosocial stress. Accordingly, to assess this question, we proposed two null hypotheses and required *both* to be rejected; (1) combined exposure does not result in a significant decrease in growth compared to *chemicals* alone; and (2) combined exposure does not result in a significant decrease in growth compared to *stress* alone. We used these criteria as follows:

Since birth weight is more naturally analyzed on the log scale, we calculated the logarithm of ratios of means. Specifically, for human and animal data on birth weight or odds ratios of low birth weight, we took the natural logarithm ratio of the mean values in the combined exposure group to the mean in each of the individual exposure groups separately: We used the following formulae to calculate the effect size, ${\bar{y}}_{ij}$;

$${\bar{y}}_{ij}=\text{log}\left(\frac{{\bar{x}}_{11}}{{\bar{x}}_{ij}}\right)$$

where *i* and *j* represent stress and chemicals respectively, and take on values of 1 (presence) and 0 (absence). Thus, the reported mean birth weight or odds ratio is represented by ${\bar{x}}_{11}$ in the combined stress and chemical group, ${\bar{x}}_{01}$ in the chemical group, and ${\bar{x}}_{10}$ in the psychosocial stress group.

We used the Delta method to transform the standard errors to the log scale and calculated a pooled standard error [42];

$$SE\left({\bar{y}}_{ij}\right)=\sqrt{{\left(\frac{SE_{11}}{{\bar{x}}_{11}}\right)}^{2}+{\left(\frac{SE_{ij}}{{\bar{x}}_{ij}}\right)}^{2}}$$

We calculated 95% CI on the natural log scale and transformed these and the effect size back to the original scale by exponentiation (using the *exp* function in Microsoft Excel (2010)). We rejected either null hypothesis if the effect size was significantly different from one (no effect).

Similar to above, we focused our second study question on fetal growth, which became:

- **Study question 2:** Is there an interaction between prenatal exposure to exogenous chemicals and stress as it affects decrements in fetal- or early-life growth?

To assess this question, we evaluated the extent to which there was a multiplicative effect of chemical exposures and stress. To do this, for each data type (e.g. odds ratio or mean birth weight), we calculated the main effects for each exposure group separately and then calculated their interaction.

For mean birth weight in humans or gestational, birth, and postnatal weights in rodents, we calculated the magnitude of the main effects on the natural log scale using the formula;

$$y_{ij}=\text{log}\left(\frac{{\bar{x}}_{00}}{{\bar{x}}_{ij}}\right)$$

where ${\bar{x}}_{ij}$ is as defined above, and ${\bar{x}}_{00}$ is the reported mean birth weight in the control group.

We used the Delta method to transform the standard errors to the log scale and calculated a pooled standard error:

$$SE\left({\bar{y}}_{ij}\right)=\sqrt{{\left(\frac{SE_{00}}{{\bar{x}}_{00}}\right)}^{2}+{\left(\frac{SE_{ij}}{{\bar{x}}_{ij}}\right)}^{2}}$$

Next we calculated the interaction term as follows:

1. When mean birthweights were available for all four groups, we were able to use the following formula on the logarithmic scale;
  Defining $z_{ij}=\text{log}\left({\bar{x}}_{ij}\right)$, the interaction term is:
  $$z_{11}+z_{00}-\left(z_{01}+z_{10}\right)$$
  The standard error of the above was calculated using the Delta method;
  $$\overset{1}{\underset{i=0}{\sum }}\overset{1}{\underset{i=0}{\sum }}{\left(\frac{SE_{ij}}{{\bar{x}}_{ij}}\right)}^{2}$$
  Finally, we calculated 95% CI on the natural log scale as described above, exponentiated the values back to the original scale and assessed whether the interaction was significantly different from one.
2. For odds ratios reported or calculated with respect to a common reference group (*x*_00_), we compared the odds ratio for the combined effect to the multiplication of the odds ratios for each effect. We calculated the magnitude of their interaction using the formulae for relative risks as described previously [43]:
  $$OR_{int}=\frac{OR_{11}}{OR_{10}\times OR_{01}}$$
  With SE calculated using the Delta method using the odds ratio on the natural log scale which we converted back to the odds ratio scale using the *exp* function in Microsoft Excel (2010).;
  $$\text{SE}\left(\text{log}\left({\text{OR}}_{\text{int}}\right)\right)=\sqrt{SE{\left(\text{log}\left(OR_{11}\right)\right)}^{2}+\;SE{\left(\text{log}\left(OR_{10}\right)\right)}^{2}+SE{\left(\text{log}\left(OR_{01}\right)\right)}^{2}}$$
  We exponentiated the main effects and interaction back to the original scale to assess whether the effects were significantly different from one (one means additive, but no interaction).
3. For data reported as odds ratios with separate reference groups (i.e. assessing chemical effects in low versus high stress groups), we took the reported odds ratios as the main effects of the chemical and combined exposures respectively. We calculated their interaction by dividing the odds ratio for the combined exposure group by the odds ratio in the chemical group.

#### Meta-analysis

We conducted a meta-analysis on the effect of individual and combined exposure to smoking and low SES on the odds of LBW. We focused on smoking studies, because studies on other chemicals were too few in number to conduct a robust meta-analysis. Specifically we used the metan function in Stata (version 11) and used a random effects model to account for anticipated heterogeneity in exposure assessment. We did not formally assess for publication bias due to the small number of included studies.

## Results

### Included studies

**Fig 1:**
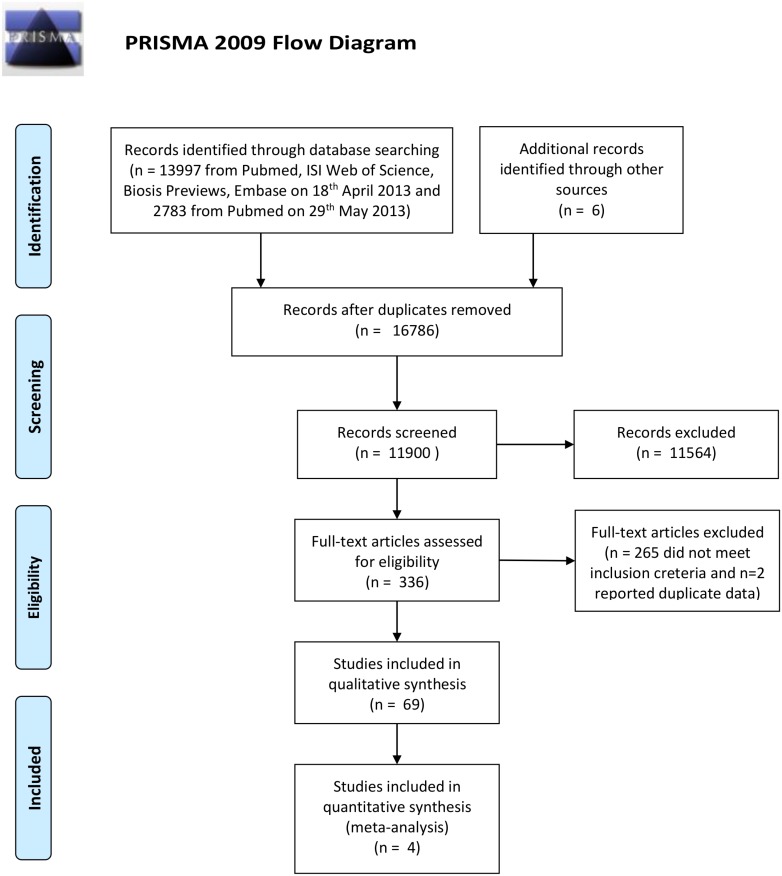
Prisma flow diagram. A flow diagram of the progression from the literature search to inclusion in the systematic review (n = 69 studies) and quantitative analysis (n = 39).

We identified 13997 studies in our initial literature search (April 2013) and a further 2783 studies from a second search of Pubmed in May 2013. In total we excluded 4886 duplicates and screened the titles and abstracts of the remaining 11894 studies. Of these, 11564 did not meet the inclusion criteria, leaving 330 publications which were included in the full text screen. A further 267 full texts were excluded based on criteria, leaving 63 relevant studies for inclusion in the systematic review. Hand searching the reference list of these studies identified an additional six relevant studies. We identified two mammalian studies which appeared to report duplicative data and thus we excluded both studies from the analyses. In total we identified 69 relevant studies reporting on the main effects of chemical and stress exposures individually and combined on any developmental outcome (Fig 1). These were comprised of 34 human studies (all full publications) and 35 animal studies (32 full publications and 3 meeting abstracts not published in full). The median year of publication was 2002 (interquartile range (IQR) 1996 to 2007). We included 17 human and 22 animal studies measuring fetal growth in our quantitative analysis of main effects and interactions (see below for details).

We identified 16 unique exogenous chemicals of which the most commonly reported were smoking, with 24 human studies [26, 29, 31–37, 44–58], and alcohol, with 3 human [32, 36, 55] and 11 animal studies [59–69] (Table 4). There were four studies each on air pollution (3 human [27, 28, 70], 1 animal [71]), lead (1 human [72], 4 animal [73–76]), and perfluorooctanesulfonic acid (4 animal [77–80]). The remaining 9 chemicals studied were reported in three or fewer studies [30, 59, 81–98]. We identified 14 measures of psychosocial stress exposures, all of which were unique to either humans or animals (7 exposure measures each). In human studies, stress exposures relating to socioeconomic status (e.g., family income, social class and education attainment level) were most common (n = 22 studies [26, 28–35, 45–47, 49, 50, 52, 56, 58, 70, 72, 82, 83, 85]), followed by race (n = 10 [26–28, 44, 48, 53–55, 57, 84]). In animal studies, restraint stress alone was the most commonly studied stress condition (n = 27 [59, 60, 62, 65–67, 69, 73–80, 86, 87, 89–91, 94–100]). The remaining five human stressors and six animal stressors were reported in three or fewer studies (Table 4).

**Table 4:** Chemical and stress exposures assessed included 69 human and animal studies on developmental outcomes from combined exposure. The sum of the number of studies for either exogenous chemical or psychosocial stress is greater than 69 because some studies reported more than one stressor variable. Numbers in parentheses indicate the number of studies which were included in the quantitative review. Under the stress/stressor exposure, “multiple stressors” refers to a random schedule administered in two rat studies consisting of exposure to each of the following stressors: mouse cage, food & water deprivation, empty water bottle, damp bedding, cage tilting, noise, crowding, alone in cage, alone in cage, no bedding and new partner [92, 93].

	Number of Studies		
Exogenous chemical exposure	Total	Human	Animal
Air pollution	4 (2)	3 (2)	1 (0)
Alcohol	14 (8)	3 (3)	11 (5)
Alcohol + methylmercury	1 (1)	0	1 (0)
Arsenic/arsenite	3 (1)	1 (0)	2 (1)
Benzene	1 (1)	1 (1)	0
Caffeine	1 (1)	0	1 (1)
Diesel exhaust	1 (1)	0	1 (1)
Aluminum	3 (3)	0	3 (3)
Lead	5 (2)	1 (0)	4 (2)
Manganese	1 (0)	0	1 (0)
Methylmercury	3 (1)	1 (0)	2 (1)
Uranium	2 (2)	0	2 (2)
PFOS	4 (3)	0	4 (3)
Smoking	24 (12)	24 (12)	0
Toluene	3 (3)	0	3 (3)
Traffic	3 (2)	3 (2)	0
Stress/stressor exposure	Total	Human	Animal
Heat	2 (1)	0	2 (1)
Life events	1 (1)	1 (1)	0
Light (no light or constant light)	2 (1)	0	2 (1)
Maternal mood	1 (1)	1 (1)	0
Mental health disorders	1 (0)	1 (0)	0
Multiple stressors	2 (2)	0	2 (2)
Nest material restriction	2 (1)	0	2 (1)
Noise	3 (0)	0	3 (0)
Occupational stress	1 (1)	1 (1)	0
Parental history of anti-social behavior	1 (0)	1 (0)	0
Race	10 (4)	10 (4)	0
Restraint	27 (18)	0	27 (18)
Restraint + bright light	1 (1)	0	1 (1)
SES, income, social class or education level	22 (12)	22 (12)	0

**Fig 2:**
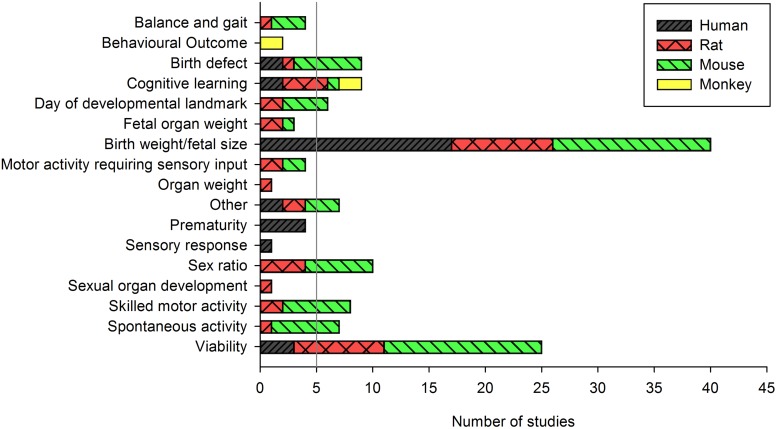
The number of studies reporting on each outcome category. The vertical gray bar represents the cut-off for the number of human studies required to take the outcome forward to a quantitative analysis.

In these studies, outcomes on growth were reported most often: 17 human studies reporting on birth weight, reduction in birth weight, odds of low birth weight or small for gestational age; and 23 animal studies reporting on fetal weight before term, at term, or postnatally. Across all human studies, we identified 6 additional groups of outcomes: viability (n = 3), prematurity (n = 4), birth defects (n = 2), cognitive learning (n = 2), sensory response (n = 1) and “other” (n = 2) which included odds of childhood obesity and probability of high physical aggression (Fig 2). Across all animal studies, measures of viability (e.g. litter size, number of resorptions) (n = 22 studies) and sex ratio (n = 10) were assessed frequently.

Due to low overlap between chemical and stress exposures reported between animal and human studies, in the following sections we have reported on these separately.

### Risk of bias for individual studies

**Fig 3:**
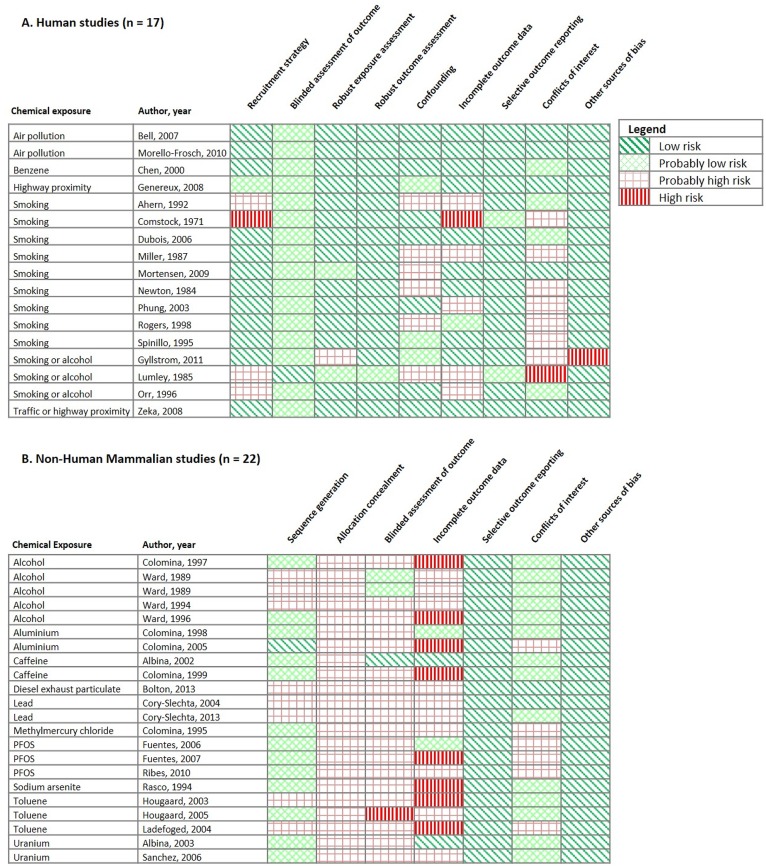
Risk of bias assessment. A heat-map of the risk of bias for human and non-human studies on growth outcomes and combined chemical and psychosocial stress.

The risk of bias assessment was conducted on fetal growth outcomes studies as these were most commonly reported with evidence amenable to quantitative assessment of combined exposure effects (Fig 3).

#### Human studies (N = 17)

In general, we found that the studies had low or probably low risk of bias across the domains (Fig 3). For example, most studies were low risk of bias for recruitment strategy (71%), exposure assessment (82%), robust outcome assessment (94%), selective outcome reporting (88%) and other sources of bias (94%). For blinded assessment of outcome we found that 6% had a low risk of bias and the remaining 94% had a probably low risk of bias. We did find more studies had a probably high risk or high risk of bias for recruitment strategy (24%), confounding (35%), incomplete outcome data (35%), and financial conflict of interest (41%). Three studies were given high risk of bias ratings for either recruitment strategy, incomplete outcome data, conflicts of interest or other sources of bias (Fig 3A).

#### Non-human mammalian studies (N = 22)

Across seven risk of bias domains, we found that studies were more likely to have probably high or high risk of bias, particularly across the domains of allocation concealment (100%), blinding (87%) and incomplete outcome reporting (82%) (Fig 3B). We found that all studies were rated low risk for selective outcome reporting and other sources of bias, and most studies were rated as low or probably low risk for sequence generation (64%) and conflicts of interest (73%).

### Summary of findings from human studies

Seventeen studies reported on measures of birth weight including birth weight itself (n = 5 studies), reduction in birth weight (n = 4), incidence of low birth weight (n = 4), odds of low birth weight (n = 5), and odds of small for gestational age (n = 1; this study also reported on odds of low birth weight).

#### Main effects of chemical exposure (low stress)

**Fig 4:**
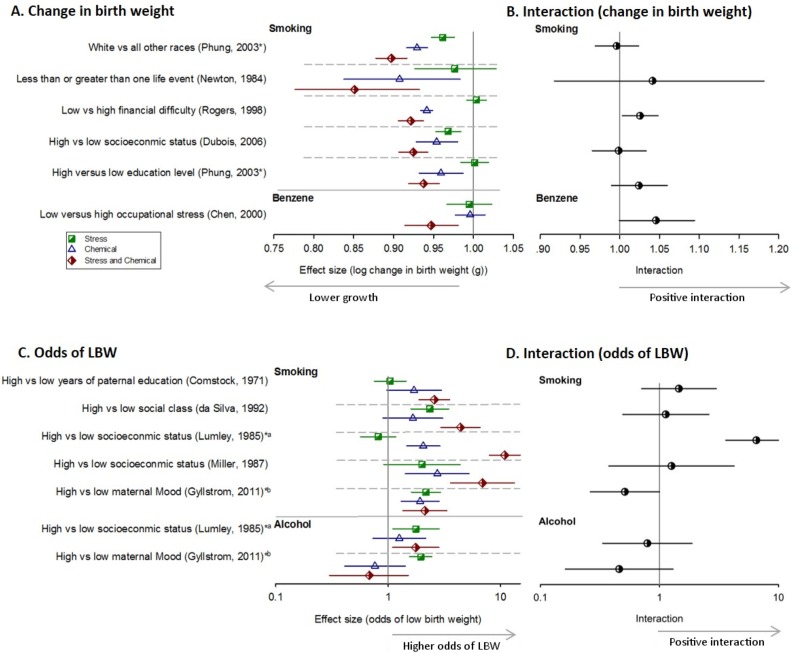
Impact of stress and chemicals on fetal development. The impact in humans of stress alone (green squares), chemicals in low stress (blue triangles) versus high stress (red diamonds) on birth weight (A) and odds of low birth weight (C) and their interaction (B and D respectively). Horizontal error bars represent 95% confidence intervals. Asterisks’ and letters denote the same population measured for more than one chemical or stress exposure.

**Fig 5:**
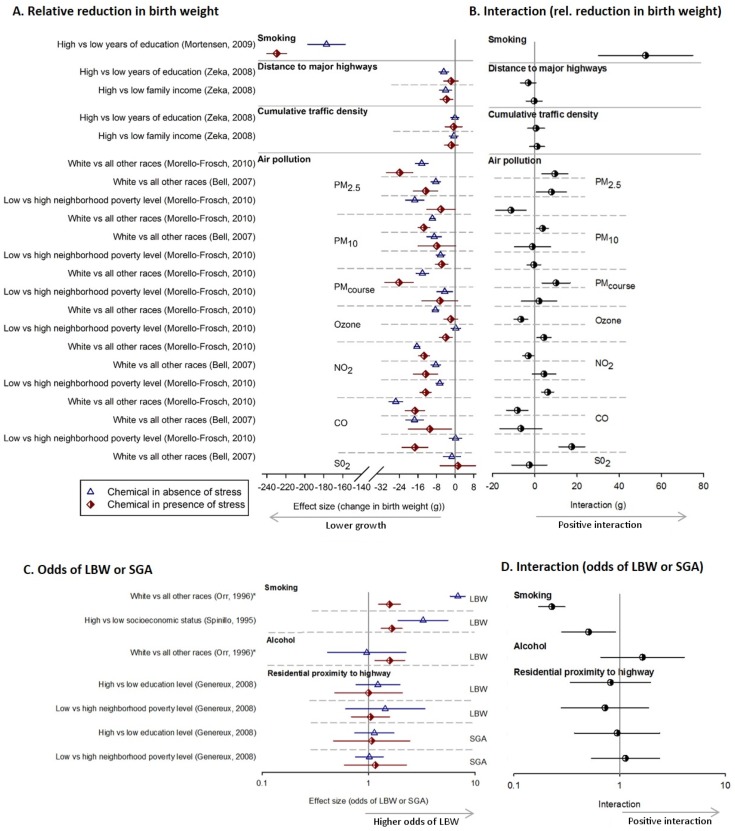
Impact of stress and chemicals on birth weight. (A) and (C) Birth weight effects in humans of chemicals in low stress (blue triangles) versus high stress (red diamonds) groups; and (B) and (D) the interaction between stress and chemicals on relative reduction in birth weight. Horizontal error bars represent 95% confidence intervals. Asterisks’ represent the same population measured for more than one chemical or stress exposure.

Human studies on fetal growth outcomes were available for 12 chemicals, of which studies on smoking was the most commonly studied (n = 12 studies). We found that smoking was associated with: a significant reduction in mean birth weight for all five studies with data on this endpoint (Fig 4A); significantly greater relative reduction in birth weight for the single study with this endpoint (Fig 5A); and significantly greater odds of low birth weight for five of seven studies for this endpoint (Fig 4C).

The relationship to birthweight for the remaining 11 chemicals/pollutants was available in 5 studies and included air pollution related exposures, benzene and alcohol. We observed significant relationships for PM_2.5_, PM_10_, PM_course_ and NO_2_. We did not observe significant relationships with benzene, alcohol, cumulative traffic density, sulfur dioxide (Figs 4 & 5).

#### Main effects of psychosocial stress exposure (low chemical)

We assessed the effects of nine broad stress exposures on mean birth weight and odds of low birth weight in humans (Table 3). We found that these stressors were associated with negative effects on birth weight, but it was not consistent (Fig 4). One study on each of race [26] and SES [46] were associated with significant reductions in mean birth weight (Fig 4A); and one study on each of social class [34] and low maternal mood were associated with significantly greater odds of LBW (Fig 4C). Two studies evaluating stress and birth weight stratified both by smoking status and alcohol status in the same sample of women but with different measures of stress; in the first study, low maternal mood was associated with significantly greater odds of LBW in both non-smokers and non-drinkers [36]; conversely in the second study [32], low SES was associated with a significant effect on odds of LBW in non-drinkers but not non-smokers (Fig 4C).

#### Combined effect of chemical and stress exposures

Smoking in the presence of high-stress was associated with a significant reduction in mean birth weight for all five different stress comparisons (Fig 4A), a significantly greater odds of LBW for all five comparisons (Fig 4C).

Of the other 11 chemicals and hazards (which were mostly air pollution related exposures), we in general found that with the exception of traffic proximity exposures (distance to major highways, cumulative traffic density and residential proximity to highway), there was a significant decrease in BW or increasing the odds of LBW in the presence of high-stress compared to the control group (low stress/low chemical) (Fig 5A and 5B).

#### Interaction

We found that the combined exposure group resulted in a significant worsening in outcome compared to either exposure alone in 6 of 13 comparisons (Figs 4A and 5A, Table 5).

**Table 5:** A summary of the effects of combined exposure to high stress and high chemical in comparison to either one alone in 10 human studies. To conclude that combined exposure is significantly associated with lower fetal growth than either exposure alone (high stress, low chemical & low stress, high chemical) we required a significant difference (denoted by “+”) between combined exposure and both individual exposures. Non-significant differences are denoted by “-“. Superscripts represent samples of the same population stratified by two chemical exposures.

				Is combined exposure significantly associated with lower fetal growth than:		
	Reference	Stress exposure	Chemical exposure	High Chemical & Low Stress	High Stress & Low Chemical	Stress or Chemical exposure alone?
Mean birth weight	[26]	Education level	Smoking	-	+	-
	[46]	Socioeconomic Status	Smoking	+	+	+
	[35]	Financial Difficulty	Smoking	+	+	+
	[37]	Life events (number of)	Smoking	-	+	-
	[26]	Race	Smoking	+	+	+
	[81]	Occupational Stress	Benzene	+	+	+
Odds of low birth weight	[54]	Socioeconomic Status	Smoking	-	+	-
	[34]	Social Class	Smoking	+	+	+
	[32]^a^	Socioeconomic Status	Smoking	-	+	-
	[31]	Paternal years of education	Smoking	+	+	+
	[36]^b^	Maternal Mood	Smoking	-	-	-
	[32]^a^	Socioeconomic Status	Alcohol	-	-	-
	[36]^b^	Maternal Mood	Alcohol	-	-	-

We found a statistically significant interaction between smoking and high financial difficulty on mean birth weight (Fig 4B) and lower social class on odds of LBW (Fig 4D). For data on relative reduction in birth weight, smoking among those with lower educational attainment resulted in a significant interaction on the original scale. The remaining interaction terms on smoking and stress were either elevated but not significant, were null, or were in the opposite direction (n = 4 for mean birth weight (Fig 4B) and n = 3 for odds of LBW; Fig 4D).

For the remaining stress and chemical exposures, their interaction was less clear; a similar number of air pollutants in the presence and absence of stress (either race or neighborhood poverty) had a significant and non-significant interaction on the original scale; we found an interaction between benzene with high occupational stress and reduced birth weight [81] (Fig 4B). We found that the interactions between alcohol and low maternal mood [36] or low SES [32] were essentially null or negative, but with confidence intervals that crossed one (Fig 4D). Interaction results for traffic proximity variables combined with stress were essentially null (Fig 5B).

#### Meta-analysis: Smoking and low SES on odds of LBW

**Fig 6:**
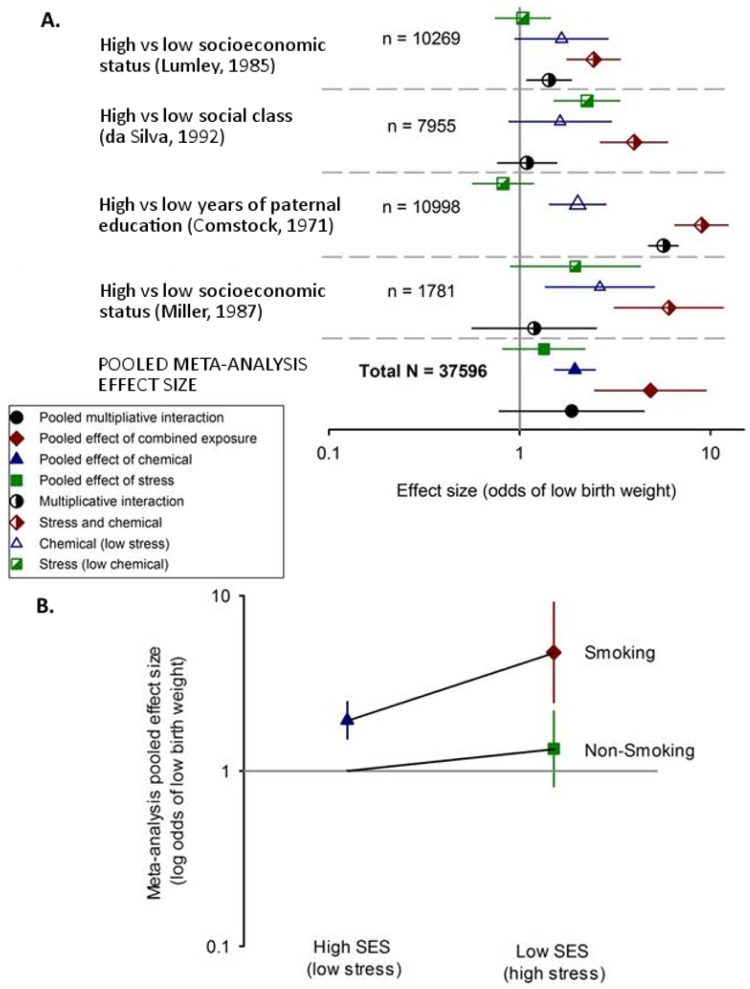
Meta-analysis of the effect of the low SES and smoking in high and low SES groups on odds of low birth weight (LBW) in humans. (A) The effects of low SES alone (high stress; green squares) or smoking in high SES (low stress; blue triangles) and low SES groups (high stress; red diamond) are shown for each of four studies and their pooled random-effects estimate at the bottom of the figure; horizontal error bars represent 95% CI, symbol sizes represents the log of the precision of the estimate (i.e. weight in the meta-analysis). (B) the random-effects pooled interaction effect between low and high SES in smokers and non-smokers. Vertical error bars represent 95% CI and the horizontal grey bar represent the line of no effect.

We identified four comparisons suitable for meta-analysis from 4 unique studies involving 31,003 human participants on the effects of smoking and stress based on SES or similar proxies for SES (years of paternal education and social class). Smoking was associated with significantly increased odds of LBW in the high stress (low SES) group (combined exposure, OR 4.75 (2.46–9.16); Χ^2^ = 33.28, I^2^ = 91.0%) compared to the low stress group (high SES) (OR 1.95 (95% CI 1.53–2.48); Χ^2^ = 1.49, I^2^ = 0%). There was also an elevated but not statistically significant odds ratio of LBW for high stress (low SES) compared to low stress among non-smokers (OR 1.34 (0.82–2.20); Χ^2^ = 16.24, I^2^ = 81.5%). The pooled interaction term from the meta-analysis was positive but not significant (1.87 (0.78–4.48); Χ^2^ = 14.25, I^2^ = 79.0%; Fig 6). Due to the small number of included studies we did not formally assess for publication bias.

### Summary of findings from mammalian studies

In the following sections we describe the data on pre- or post-natal offspring weight from 22 studies with quantitative data amenable to analysis. Six studies reported only behavioral outcomes [61, 63, 64, 75, 79, 98]; three meeting abstracts did not report quantitative data [60, 71, 96]; three studies reported only qualitative information on fetal growth [62, 69, 76] and one study did not report the sample size [59]. The studies with quantitative data that we analyzed had a median average sample size per group of 10 litters across the four exposure groups (IQR 9 to 12). The effect sizes for the combined and individual exposures to chemicals and stress and their interaction are presented in the S1–S6 Figs. Note that the majority of studies reported more than one comparison from the same cohort of animals, representing multiple time points of assessment.

#### Main effects of chemical exposure

**Fig 7:**
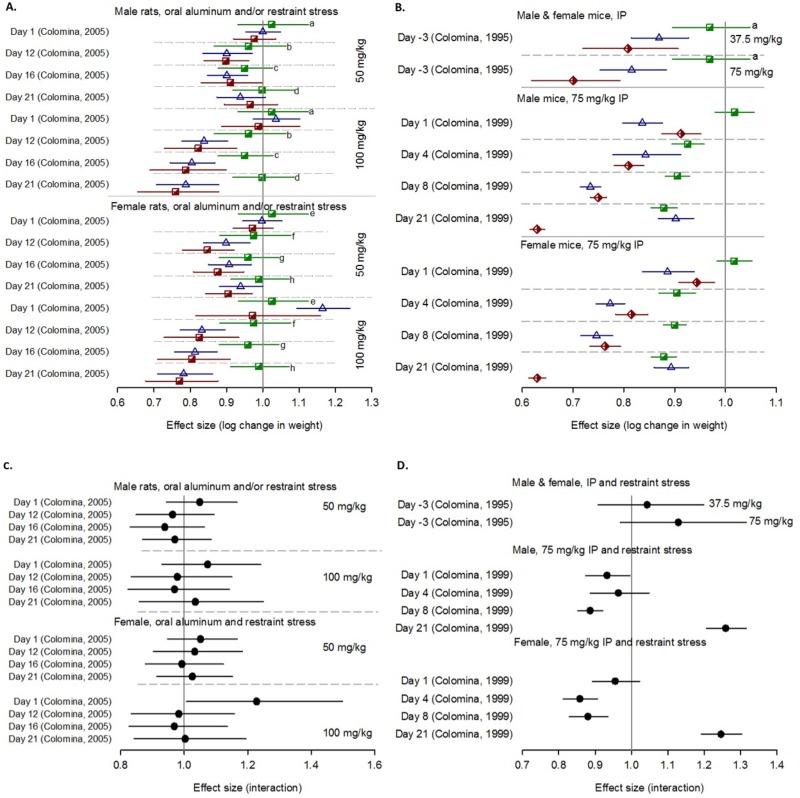
The effect of prenatal aluminum and restraint stress in rats and mice. The impact of stress alone (green squares), chemicals in low stress (blue triangles) versus high stress (red diamonds) on postnatal weight at various times of assessment in rats (A) and mice (B) and their interaction (C and D respectively). Horizontal error bars represent 95% confidence intervals; the vertical grey bar represents the line of no effects. Letters denote the same stress control group used for multiple doses at the same time point. Figure legend is the same as Fig 4. Abbreviation: IP, intraperitoneal.

We assessed ten chemicals quantitatively (alcohol (n = 5 studies), aluminum (n = 3), caffeine (n = 1), diesel exhaust particulates (n = 1), lead (n = 2), methylmercury chloride (n = 1), PFOS (n = 3), sodium arsenate (n = 1), toluene (n = 3) and uranium (n = 2)). We found a negative association between prenatal aluminum exposure and BW at all but the earliest time points of assessment (postnatal day 1 in rats (1 study, 16 comparisons; Fig 7A) and gestational day 15 in mice (2 studies, 10 comparisons); Fig 7B). Additionally we found a trend towards weight reduction by alcohol in both rats (n = 2 studies, 3 comparisons) and mice (n = 3 studies, 8 comparisons (S1 Fig)). For all other chemicals, the effects were unclear and the vast majority of comparisons had wide confidence intervals that crossed the line of no effect (S2 to S6 Figs).

#### Main effects of stress exposure

We assessed six methods of exposing animals to stress (restraint (n = 18) heat (n = 1), nest material restriction (n = 1), no light (n = 1), restraint with bright light (n = 1), and various stressors (n = 2)) (Table 4). Although the vast majority of studies used restraint stress, there was little consistency in findings, albeit with a slight trend towards a reduction in weight, and the majority had confidence intervals which crossed the line of no effect. For all studies the time of administration began during pregnancy but the time of assessment of weight varied widely (from 3 days prior to birth until day 90 in mice; and from 1 day prior to birth until day 23 in rats), and most studies reported weight at multiple time points. We pooled restraint-stress data from these different times of assessment for mice and rats in a meta-analysis and found nine of ten summary estimates were negative (inferring a reduction in growth due to restraint stress); however for all but one comparison, the confidence intervals crossed the line of no effect (S1–S6 Figs).

#### Combined effect of chemical and stress exposures

Of the ten chemicals we evaluated quantitatively, there was a significant reduction in fetal growth outcomes when we evaluated prenatal aluminum exposure and prenatal restraint stress in mice combined at all times of assessment and rats at all but the earliest times points and one comparison measured at postnatal day 21 (Fig 7). Additionally we found a trend towards a reduction in weight when toluene (assessed in two studies with ten comparisons) was paired with restraint or a variety of stressors (data not shown). For all other chemical and stress exposures there was no clear trend.

#### Interaction

In 98 comparisons from four studies, we found five comparisons where the combined exposure to stress and chemicals was associated with significantly lower weight than either exposure alone; these were for toluene and various stressors, aluminum and restraint (3 comparisons, 2 studies) and sodium arsenite and restraint. For chemicals including alcohol, PFOS, lead and uranium we found no evidence of a negative combined exposure. We found a trend towards positive interactions for toluene paired with restraint or various stressors in rats; however, all but one comparison had wide confidence intervals which crossed the line of no effect. Interestingly we found a trend towards negative interactions (albeit most comparisons were not significant) for both alcohol paired with restraint, heat or light in mice and uranium paired with restraint in rats. For all other chemicals and stressors, there was no consistent trend.

## Discussion

In this first systematic review on the combined impact of prenatal exposure to environmental chemicals and psychosocial stress on fetal and developmental outcomes, we found only a small body of evidence on this topic with substantial variation between studies in terms of the individual and combined exposures investigated. However, in general we found human evidence of an association between chemical exposure and reduced birth weight, and some evidence for an association between increased stress and reduced birth weight. We found evidence for a combined effect of chemical exposure and stress on birth weight, as this was statistically significant in our meta-analysis of smoking and stress. The interaction term was positive, but not statistically significant.

There has been increasing attention to the interaction between environmental chemical exposures and chronic psychosocial stress as having a cumulative effect on health outcomes, and on whether this potential interaction may, in part, explain observed health disparities [22]. However, our systematic review, at least for developmental exposures, finds that the literature is sparse. We identified nearly 14,000 potential studies in our search, but very few of these covered the topic of combined evaluation of environmental chemical exposures and stress. A further limitation is that multiple exposures and outcomes were measured; further limiting the evidence on any one topic. For example, for most outcomes we found 5 or fewer studies, with the exception of birth weight and viability. Our search is unlikely to be biased or incomplete, as we did a double screen on ten percent of the literature search results and found no discrepancies in the findings.

Below, we summarize the findings for human and nonhuman evidence and highlight the strengths and weaknesses of our approach and the available evidence.

### The impact of individual or combined exposures in humans

Across human studies we found consistent evidence of an association between higher chemical exposure and reduced birth weight. This trend was less consistent for stress exposures and reduced birth weight and in our analyses several comparisons were not significant. However, when exposed to both high levels of stress and chemicals, we found that birth weight was consistently reduced compared to the control group.

A further factor that could decrease our ability to observe relationships is that we dichotomized each exposure into “high” and “low” in order to report the findings from each study on the same scale; however this approach may have diluted some effects. For example we considered any tobacco exposure, whether it be passive smoking or heavy smoking under the category of “high” exposure. For each variable the co-authors discussed the most conservative cut-offs *a priori*. We attempted to perform a sensitivity analysis on the smoking data by looking at the effects of the highest reported category of exposure versus the lowest; however only two of eleven studies reported multiple exposure levels; the remaining nine studies dichotomized into two exposure groups which we used for our analyses.

Finally, stress exposures are difficult to measure and were measured variably across the human studies; stress exposure can be broadly grouped into place-based stress (clusters of high stress conditions such as neighborhoods of greater poverty) and individual-based (e.g. discrimination and number of life events). Some of these exposures are direct and others are indirect; however in the present study we considered all exposures equally, including exposures which can be classified as indicators of stress response (e.g. maternal mood) and stressors (e.g. SES). Additionally, we found little methodological consistency across studies in their measurement of chronic psychosocial stress.

### Smoking and socioeconomic status

Given the limited evidence, our meta-analysis only included a subset of the data and only on one type of chemical exposure (smoking in four studies) which we considered sufficiently homogeneous for a meta-analysis; however the meta-analysis is likely to be underpowered due to the small number of included studies. Nevertheless, our meta-analysis results on the effect of smoking and low SES, found a clear trend towards a reduction in birth weight for smoking in both the absence and presence of low SES. Our data on the effects of smoking confirm findings from earlier studies, including a meta-analysis of environmental tobacco exposure which identified significantly higher odds of LBW in the exposed group [101]. Moreover, the effect was stronger in the presence of low SES. In contrast, low SES (a surrogate for higher stress exposure) in the absence of smoking had no effect on birth weight and the interaction term was elevated but not significant. Interestingly, a previous study on the effects of socially ascribed status (in this case gender, race and ethnicity) in combination with smoking showed an additive effect on mortality, whereas achieved status (including educational attainment, labor force participation, and occupation) had an interaction with smoking on morbidity [102].

### Evidence from non-human mammalian studies

In general the findings from animal studies were not consistent; however, the dataset was extremely heterogeneous and sparse. Of the nine chemical exposures, only aluminum had a consistent effect, with weight reductions at all but the earliest time points in mice and rats; however, these data were from 3 studies (2 on mice and 1 on rats) with multiple time points of assessment and so we were unable to pool the data in a meta-analysis. We also found evidence of weight reduction by alcohol in rats; however, these data were comprised of three comparisons from just two studies, and the effects in mice were inconsistent. We also found that uranium was linked to a reduction in weight with later times of assessment; however, the majority of comparisons (16 of 18) were from two cohorts of animals from a single study.

Although a more consistent measure of stress exposure was used across studies, with the majority applying restraint stress, we generally did not find a trend in the relationship between stress and fetal growth. However, pooling the data in a meta-analysis at different time points, we found that restraint stress was modestly associated with reduced weight, but the data were only significant at one range of time points (postnatal days 6 to 9).

When animals were exposed to both the chemical and stress, we found a trend towards a relationship between aluminum, restraint stress and reduction in weight for mice at all times points; for rats we observed effects at all but the earliest time points, for toluene and restraint or various stressors, and uranium and restraint.

Interestingly, we did not find clear interactions for any of the pairs of exposures although there was a trend towards positive interactions for alcohol in mice. We also found a trend towards negative interactions for toluene paired with restraint or various stressors and uranium paired with restraint stress, indicating that there was not greater weight loss in the combined exposure group compared to the chemical and stress groups.

Finally, for four of the chemicals (caffeine, diesel exhaust particulate, methylmercury chloride and sodium arsenate), the number of comparisons were too small to draw meaningful conclusions.

### Limitations

There are a number of potential limitations to our systematic review. First, despite our attempt to conduct a robust and comprehensive literature search, we cannot be certain that we identified every relevant study. However, we performed a snowball search to try to reduce this risk. In addition, due to the heterogeneity between both human and animal studies, we were unable to formally assess for potential publication bias. Second, the limited number of studies, and the breadth of the research question meant we were only able to perform a meta-analysis on one combination of stress and chemical exposure which was in itself only based on four studies. Third, although we set out to follow the Navigation Guide methodology, we did not perform the fourth step—rating the body of evidence to make a final recommendation on the association between the exposures and outcome. This is because our primary objective was to assess the scope of the literature and in performing a review of such a broad topic, any combination of chemical and stress exposure, we did not believe this step to be feasible. Making a formal recommendation on the body and strength of evidence is better suited to a single exposure with inherently less heterogeneity in measurement approaches. Finally, we attempted to qualitatively assess the quality of the relevant studies using the risk of bias tool outlined in the Navigation Guide.

### Recommendations

There has been increasing attention to the interaction between environmental chemical exposure and chronic psychosocial stress as having a cumulative effect on health outcomes, and on whether this potential interaction may, in part, explain observed health disparities [103]. However, our systematic review, at least for developmental exposures, indicates that the literature is sparse and too inconsistent to make firm conclusions about the presence of interactions. There are several key areas of research needed to advance our understanding of interactions between chemical exposure and chronic psychosocial stress: 1) develop and apply more consistent measures that reflect the different dimensions of chronic psychosocial stress in human epidemiologic studies; 2) evaluate the methods used for inducing chronic psychosocial stress in animal studies to ensure they are reflective of stress in humans; and 3) expand the evaluation of combined chemical exposures. These recommendations are based on the following limitations we faced in our systematic review. First, there are a diverse array of stress exposures of relevance to fetal growth and developmental outcomes, which pose significant challenges to utilizing consistent measure of stress across studies; moreover, there are key differences across studies in terms of whether stress is measured at the individual- or community-level. For example, the stress of living in a high poverty neighborhood compared to a low poverty neighborhood may not exert the same adverse effect as individual-level measures of high versus low socioeconomic status or material deprivation. Although it is challenging to apply uniform approaches to measuring stress exposure due to its diverse dimensions, efforts to harmonize more commonly used measures, such as those related to individual-level poverty or neighborhood level socioeconomic status, could better facilitate future systematic reviews to assess the combined effects of stress and chemical exposure. Our capacity to systematically assess combined effects in human studies is further complicated by the lack of consistent chemical exposures among studies that evaluate combined effects with chronic psychosocial stress. Second, while the animal studies had a more consistent approach to measuring stress (restraint stress), the literature indicates that this may not sufficiently reflect chronic psychosocial stress in humans [104]. Further, there was an insufficient number of studies to evaluate the combined effects of chemicals and restraint among animals during development.

## Conclusions

To our knowledge this is the first systematic review and meta-analysis on the effects of cumulative prenatal exposure to stress and environmental chemicals on developmental outcomes. Overall we found this to be an understudied topic and recommend further investigation in high quality, pragmatically designed animal or human observational studies. However, within the context of the small body of evidence currently available, we found that exposure to several chemicals in the absence of stress is associated with a reduction in fetal growth in humans, and this effect is generally larger in the presence of stress. Conversely, we found little effect of stress in the absence of chemical exposure. Our assessment of the animal data were limited by the small number of studies on each chemical and stressor and by limitations to their quality, particularly the lack of allocation concealment, sequence generation and blinding. Overall, we found that prenatal exposure for several chemicals is harmful, but there is limited evidence of interaction with stress exposure, and combined exposure was associated with significantly reduced fetal weight in comparison to either exposure alone in about half of studies.

## Supporting information

S1 Table The PRISMA 2009 checklist. (DOC)

S1 Fig The effect of prenatal alcohol and/or stress on fetal growth. Figure legend is the same as Fig 7. Superscripts a and b refer to citations [58] and [59] respectively.
(EPS)

S2 Fig The effect of prenatal lead and/or stress on fetal growth. Figure legend is the same as main Fig 7.
(EPS)

S3 Fig The effect of prenatal other chemicals (see graph labels) and/or stress on fetal growth. Figure legend is the same as Fig 7.
(EPS)

S4 Fig The effect of prenatal PFOS and/or stress on fetal growth. Figure legend is the same as Fig 7.
(EPS)

S5 Fig The effect of prenatal toluene and/or stress on fetal growth. Figure legend is the same as Fig 7.
(EPS)

S6 Fig The effect of prenatal uranium and/or stress on fetal growth. Figure legend is the same as Fig 7.
(EPS)
